# Pressure-induced posterior gastric perforation in a patient with severe gastroparesis and prior Nissen fundoplication

**DOI:** 10.1093/jscr/rjag677

**Published:** 2026-07-31

**Authors:** Jordan De Guzman, Donald Morin, Atharva Joshi, Shabnam Yazdanpanah, Paraskevi Orfanou

**Affiliations:** American University of the Caribbean School of Medicine, Florida, Miramar, 33025, United States; Department of Surgery at Henry Ford Providence Hospital, 16001 W 9 Mile Rd, Michigan, Southfield, 48033, United States; American University of the Caribbean School of Medicine, Florida, Miramar, 33025, United States; American University of the Caribbean School of Medicine, Florida, Miramar, 33025, United States; Department of Surgery at Henry Ford Providence Hospital, 16001 W 9 Mile Rd, Michigan, Southfield, 48033, United States

**Keywords:** gastroparesis, gastric perforation, gastric dilatation, Nissen fundoplication, damage-control surgery

## Abstract

Gastric perforation is a life-threatening emergency most associated with peptic ulcer disease. In patients with altered foregut anatomy and impaired motility, alternative mechanisms may occur. We describe a non-verbal 27-year-old man with a history of esophageal atresia repair, severe gastroparesis, prior Nissen fundoplication, and gastrostomy tube dependence who presented with progressive abdominal distention and acute hypoxic respiratory failure. On presentation, he was tachycardic and tachypneic with significant metabolic derangements. Imaging demonstrated pneumoperitoneum and gastric perforation. Emergent exploratory laparotomy revealed a large posterior gastric defect measuring ~8–10 cm with significant contamination, requiring partial gastrectomy and staged damage-control management. A venting gastrostomy and feeding jejunostomy were placed at re-exploration, and the patient recovered following surgical and critical care management. This case demonstrates that impaired gastric decompression can lead to progressive overdistention and perforation, resulting in emergent surgical intervention.

## Introduction

Gastric perforation is a life-threatening surgical emergency most commonly associated with peptic ulcer disease and typically presents with acute abdominal pain and peritonitis [1]. In patients with altered foregut anatomy and impaired gastric motility, alternative mechanisms of perforation may occur. We report a patient with severe gastroparesis, prior Nissen fundoplication, and gastrostomy tube dependence who developed massive posterior gastric perforation secondary to progressive gastric overdistention.

## Case report

A 27-year-old man with a complex medical history, including premature birth, bronchopulmonary dysplasia requiring tracheostomy, repaired esophageal atresia with gastrostomy tube dependence, severe gastroparesis, prior Nissen fundoplication, and cognitive impairment, presented with progressive abdominal distention and increased work of breathing. History obtained from the patient's caregiver revealed three days of progressive abdominal distention and worsening hypoxia.

On arrival, he was tachycardic, tachypneic, and hypoxic. Physical examination revealed marked abdominal distention. Laboratory evaluation (Table 1) demonstrated metabolic derangements, including lactic acidosis, hypokalemia, and acute kidney injury, without leukocytosis.

**Table 1:** Laboratory values on admission.

	Value	Units	Reference range
WBC	8.11	K/mcL	4.00–11.00
Hemoglobin	20.8	g/dL	13.5–17.5
Hematocrit	64.9	%	41.0–53.0
Creatinine	1.5	mg/dL	0.6–1.2
Sodium	140	mmol/L	136–145
Potassium	2.9	mmol/L	3.5–5.1
Chloride	97	mmol/L	98–107
Bicarbonate (CO₂)	15	mmol/L	22–29
Anion Gap	28		7–17
Lactate (serum)	9.4	mmol/L	0.5–2.0
Lipase	2710	U/L	0–160
pH	7.17		7.35–7.45
PCO₂	52	mmHg	35–45
HCO₃ (ABG)	19	mmol/L	22–26
Lactate (ABG)	6.1	mmol/L	0.5–2.2

Chest radiography demonstrated free air beneath both hemidiaphragms. Computed tomography (CT) revealed massive gastric distention with intraluminal debris and extensive pneumoperitoneum, concerning for gastric perforation (Fig. 1). Additional findings included portal venous gas, pneumatosis intestinalis, and diffuse pulmonary opacities (Fig. 2). The patient underwent emergent exploratory laparotomy for suspected gastric perforation and sepsis.

**Figure 1:**
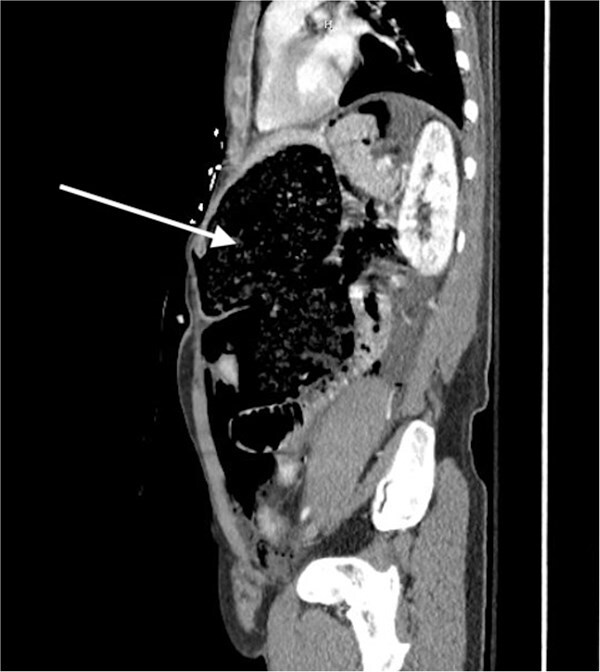
Contrast-enhanced CT demonstrating gastric distention and pneumoperitoneum demonstrating marked gastric distention with retained intraluminal contents. White arrows indicate the distended stomach. Extensive pneumoperitoneum is present, consistent with perforation of a hollow viscus. The posterior gastric wall appears poorly defined, with intraoperative confirmation of a large posterior gastric defect.

**Figure 2:**
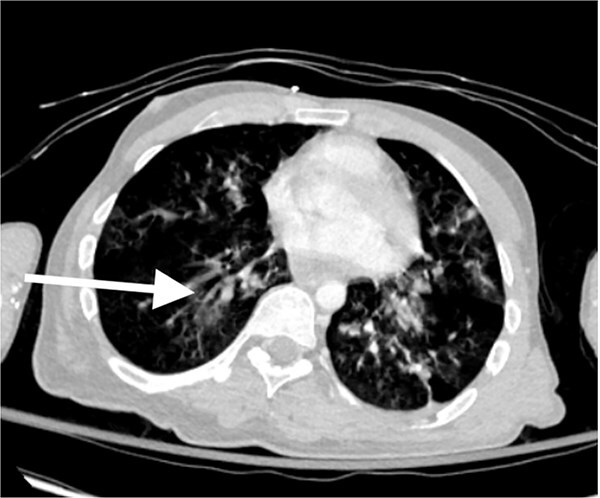
Chest CT demonstrating bilateral pulmonary opacities. Axial contrast-enhanced CT image of the chest demonstrating bilateral opacities and tree-in-bud nodularity (arrow), consistent with an inflammatory or infectious process.

Intraoperatively, extensive contamination with undigested gastric contents was noted throughout the peritoneal cavity. The stomach was densely adherent to the anterior abdominal wall, requiring adhesiolysis and removal of the existing gastrostomy tube for mobilization. A large posterior gastric perforation measuring ~8 cm was identified. Given the size of the defect and nonviable surrounding tissue, partial gastrectomy was performed for source control.

A transverse colon mesenteric defect was identified adjacent to the perforation, though the colon remained viable on Doppler assessment. Due to hemodynamic instability requiring vasopressor support, temporary abdominal closure with negative pressure therapy was performed.

The patient was admitted to the intensive care unit, where he demonstrated clinical improvement and returned to the operating room on postoperative Day 3 for re-exploration. The gastric repair was intact. The colon remained viable, confirmed with indocyanine green fluorescence imaging. A venting gastrostomy tube and feeding jejunostomy tube were placed. Due to attenuated fascia, bridging mesh and negative pressure therapy were used for closure (Fig. 3).

**Figure 3:**
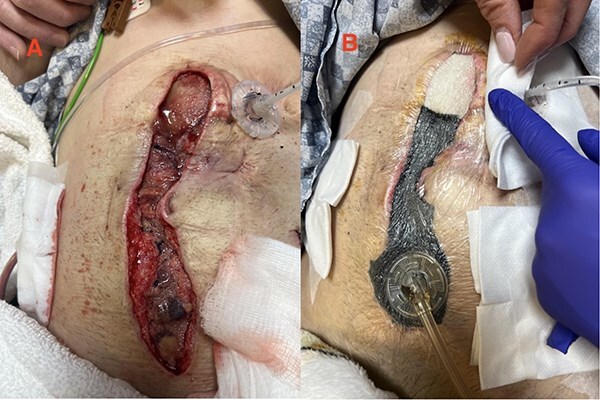
Abdominal wall management with bridging mesh and negative pressure therapy. (A) Intraoperative view demonstrating failure of primary fascial reapproximation along the superior incision site due to attenuated fascia. (B) Postoperative view demonstrating placement of a bridging absorbable mesh with negative pressure wound therapy, consistent with a damage-control approach with long-term abdominal wall reconstruction in mind.

He was gradually weaned from ventilatory and vasopressor support. Enteral nutrition was initiated via jejunostomy and advanced to goal. Serial imaging demonstrated interval gastric decompression (Fig. 4). He was discharged home with enteral feeding support and scheduled venting via gastrostomy tube.

**Figure 4:**
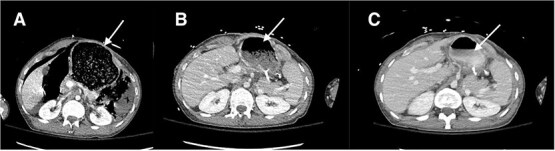
Serial CT imaging demonstrating interval resolution of gastric distention following surgical management. (A) Preoperative axial contrast-enhanced CT demonstrating marked gastric distention with heterogeneous intraluminal contents (arrow) and associated pneumoperitoneum consistent with perforation of a hollow viscus. (B) Postoperative Day 11 axial CT demonstrating interval decrease in gastric distention. (C) Postoperative Day 15 axial CT demonstrating continued reduction in gastric size, consistent with successful decompression and clinical improvement.

## Discussion

Gastroparesis is characterized by delayed gastric emptying and commonly presents with nausea, vomiting, and abdominal distention. Gastric perforation secondary to severe gastric distention is rarely reported. In this patient, severe gastroparesis impaired antegrade emptying, while prior Nissen fundoplication limited retrograde decompression through vomiting, creating a functional closed-loop physiology. The resulting rise in intragastric pressure likely exceeded mucosal perfusion pressure, leading to ischemia, necrosis, and eventual perforation [2–6]. The posterior location of the defect, absence of traditional ulcer risk factors, and marked gastric distention support a pressure-induced ischemic mechanism rather than primary peptic ulcer disease.

A period of localized containment within the lesser sac likely explains the delayed onset of diffuse peritonitis and progressive clinical deterioration. Posterior gastric perforations are known to present insidiously due to initial confinement within the lesser sac, with worse outcomes associated with delayed recognition [7]. Intraoperative identification of a large transverse colon mesenteric defect adjacent to the perforation further supports this mechanism. Although the colon remained viable, the adjacent mesentery, lacking the protective mucus barrier of gastric epithelium, may have been exposed to acidic and enzymatic gastric contents, resulting in localized tissue injury and eventual breakdown of containment [8]. Subsequent spillage into the peritoneal cavity likely led to rapid progression to diffuse peritonitis and clinical decline [9].

Acute hypoxic respiratory failure contributed to a misleading clinical presentation. Massive gastric distention and pneumoperitoneum can impair ventilation by restricting diaphragmatic movement [10]. Combined with the systemic inflammatory response of sepsis [11], these effects likely contributed to the patient's profound hypoxia and may have diverted attention from the underlying intra-abdominal pathology. In medically complex or nonverbal patients, respiratory compromise may therefore be a prominent manifestation of an abdominal catastrophe.

The presence of a gastrostomy tube highlights an important limitation: enteral access should not be assumed to provide reliable gastric decompression. Gastrostomy tube dysfunction is well documented, with potential contributors including occlusion by food debris or secretions, inadequate venting, or intragastric volume and pressure exceeding the tube’s capacity to decompress [12]. At re-exploration, the surgical team placed a dedicated venting gastrostomy and separate feeding jejunostomy, allowing decompression and nutrition to be managed independently, an approach supported in patients with severe gastroparesis [13, 14].

This patient’s surgical management reflects a damage-control approach in the setting of severe intra-abdominal sepsis and hemodynamic instability. Given the large posterior gastric perforation and extensive contamination, definitive repair was deferred in favor of rapid source control with partial gastrectomy and temporary abdominal closure, allowing for physiologic stabilization prior to re-exploration. At second-look laparotomy, the repair was intact, and adjunctive measures, including reinforcement of the staple line and placement of a venting gastrostomy and feeding jejunostomy, were performed to optimize long-term management. Primary fascial reapproximation was not feasible due to attenuated fascia, necessitating the use of bridging absorbable mesh with negative-pressure wound therapy and acceptance of a planned ventral hernia. This approach reflects established damage-control principles, prioritizing physiologic recovery over immediate anatomic closure in critically ill patients [15].

This case highlights a rare and catastrophic complication of severe gastroparesis in the setting of prior Nissen fundoplication, resulting in pressure-induced massive posterior gastric perforation—a presentation not previously described in literature. It underscores the critical role of impaired gastric decompression, where the combined effects of foregut dysmotility, altered anatomy, and ineffective venting can lead to progressive gastric distention and ischemic injury. Additionally, the presence of a gastrostomy tube should not be assumed to provide adequate decompression in high-risk patients. Timely surgical intervention and a damage-control approach are essential to improving outcomes.
